# Current Experimental Studies of Gene Therapy in Parkinson's Disease

**DOI:** 10.3389/fnagi.2017.00126

**Published:** 2017-05-03

**Authors:** Jing-ya Lin, Cheng-long Xie, Su-fang Zhang, Weien Yuan, Zhen-Guo Liu

**Affiliations:** ^1^Department of Neurology, Xinhua Hospital Affiliated to the Medical School of Shanghai JiaoTong UniversityShanghai, China; ^2^Department of Neurology, The first Affiliated Hospital of Wenzhou Medical University, Wenzhou Medical UniversityWenzhou, China; ^3^School of Pharmacy, Shanghai JiaoTong UniversityShanghai, China

**Keywords:** Parkinson's disease, animal models, gene therapy, RNA interference, neurodegeneration

## Abstract

Parkinson's disease (PD) was characterized by late-onset, progressive dopamine neuron loss and movement disorders. The progresses of PD affected the neural function and integrity. To date, most researches had largely addressed the dopamine replacement therapies, but the appearance of L-dopa-induced dyskinesia hampered the use of the drug. And the mechanism of PD is so complicated that it's hard to solve the problem by just add drugs. Researchers began to focus on the genetic underpinnings of Parkinson's disease, searching for new method that may affect the neurodegeneration processes in it. In this paper, we reviewed current delivery methods used in gene therapies for PD, we also summarized the primary target of the gene therapy in the treatment of PD, such like neurotrophic factor (for regeneration), the synthesis of neurotransmitter (for prolong the duration of L-dopa), and the potential proteins that might be a target to modulate via gene therapy. Finally, we discussed RNA interference therapies used in Parkinson's disease, it might act as a new class of drug. We mainly focus on the efficiency and tooling features of different gene therapies in the treatment of PD.

## Introduction

### Treatment for PD

With the improvement of the medical care, people enjoyed a longer life span, but it also bring about aging problems. The increasing healthy costs and the prevalence of Parkinson's disease (PD) are becoming more severe in modern society (von Campenhausen et al., 2009). But PD is so complicated that treating PD is to treat a moving target, as the disease progressed, one therapy could not solve all the problems (Simonato et al., 2013; Kakkar and Dahiya, 2015).

Treatment for PD can be classified as 3 different types: pharmacotherapy, functional neurosurgery, transplantation and gene therapy.

### Pharmacotherapy for Parkinson's disease

Among the treatments mentioned above, pharmacotherapy is the most effective one in the early years of PD (Buttery and Barker, 2014). Pharmacotherapy can be divided into two kinds: dopaminergic and non-dopaminergic way. The useing of dopamine receptor agonists, catechol-O-methyltransferase (COMT) and monoamine oxidase (MAO) inhibitors were classified as dopaminergic treatment for PD (Fang et al., 2015; Sharma et al., 2015). Using of COMT and MAO inhibitors can reduce the motor fluctuations of patients. The MAO inhibitors prolonged the duration of L-dopa action time (Muellner et al., 2015; Sari and Khalil, 2015). But with the disease advancing and the appearance of L-dopa-induced dyskinesia (LID), these therapies shows their limitations (Behari and Singhal, 2011). In order to address the problems, sustained or controlled release drugs are developed to therapy the PD (Ren et al., 2011; Yang et al., 2012a,b; Xie et al., 2014). The non-dopaminergic drug amantadine is a N-methyl-D-aspartate (NMDA) receptor antagonist that have mild symptomatic benefits and can decrease LID in a proportion of patient (Paquette et al., 2012; Rascol et al., 2015), other non-dopaminergic drug like antagonist of metabotropic glutamate receptors (mGluRs) is still under animal experiment and clinical trials (Johnson et al., 2009; Pahwa et al., 2015). Functional neurosurgery was used in advanced PD, including deep brain stimulation and lesion (Rowland et al., 2016; Verhagen Metman et al., 2016). Surgical treatment for PD can be achieved by pallidotomy and globus pallidus internus (GPi) deep brain stimulation (DBS) and subthalamotomy and subthalamic nucleus deep brain stimulation (DBS) (Poortvliet et al., 2015; Hamani et al., 2016). The GPi surgery has a direct effect on dyskinesia while the subthalamic nucleus (STN) deep brain stimulation (DBS) has a benefit on reducing the dopaminergic drug dose (Munhoz et al., 2014; Poortvliet et al., 2015).

But PD is far more complicated than commonly appreciated. As the disease progressing, the effectiveness of the drugs declined, it became incapable for patients to control the motor symptoms (Olanow et al., 2009; Bartus et al., 2014). Thus, the nigrostriatal-mediated motor impairment still lacked an adequate solution.

## Delivery method of gene therapy

The advantage of gene therapy was that we can deliver a gene as an agent to the specific brain region to alter function and treat PD (Stayte and Vissel, 2014), while avoiding the off-target effects (Allen and Feigin, 2014). Although gene therapy is mainly experimental at present, the promising future makes a lot of researchers seeing it as a new class of drugs for PD. Before we review the gene therapies for PD, we should pay some attention to the delivery method of gene therapy first (Oertel and Schulz, 2016).

While the measures we adopted to fix PD needs a lot of considerations, the measures themselves are limited by a lot of factors, for example, the chose of the delivery vector. Basically, we can divide the delivery method into two types: viral and non-viral mediates ways (Latchman and Coffin, 2001; Muramatsu et al., 2002; Lewis et al., 2014).

### Viral vectors mediate delivery methods

When we talk about typical virus vector, we indicate adeno-associate virus, retroviruses and adenovirus. Adeno-associate virus (AAV) is the most utilized vector in brain (Mochizuki et al., 2002; Fischer et al., 2016). The recombinant adeno-associate virus vector (rAAV) remains the essence of the wild-type AAV. Different subtype of AAV could enter different host-cells due to its capsid structure (Burger and Nash, 2016). AAV mainly transduces cells of nervous system, neurons, astrocytes and part of the microglia. The AAV will not integrated to host-cells, so it might dilution due to cell division. The sending of the vectors can be divided into stereotaxic injection, ventricular delivery and systematic delivery, which often require special equipment. Production of rAAV is labor intensive (Benskey and Manfredsson, 2016a).

The retroviruses include lentivirus and non-lentivirus (Benskey and Manfredsson, 2016b). They were characteristic for the ability to integrate with the host cells, thus it'll not be dilute while the host-cells dividing (Nasri et al., 2014). Compared with the AAV, The retroviruses can carry more gene fragments, nearly 9 kb. The difference between lentivirus and non-lentivirus is that the later cannot traverse the nuclei membranes, it impact actively dividing cells only, while lentivirus impact both dividing and non-dividing cells. And the retrovirus could transfect almost all kinds of cells in nervous system, the production of them is much more easier than AAV (Kumar and Woon-Khiong, 2011; Kobayashi et al., 2016).

Recombinant adenovirus (rAd) had reduced the cytotoxicity and immunogenicity to an acceptable extent. It could carry about 35 kb of genetic material. The large capacity has its own merits and drawbacks (Uil et al., 2011), for it'll require a certain mineral amount of genes to keep the optimal packaging efficiency. The rAd still have certain immunogenicity although this will not impact the transgene expression. Furthermore, the production of rAd is time consuming (Suzuki et al., 2011). Table 1a is the sum of virus vector mediates gene therapy.

**Table 1A:** **Virus vector mediates gene therapy**.

**Virus**	**Capability**	**Delivery method**	**Limitation**
Adeno-associate Virus	4.7 ~ 5 kb	Stereotaxic injection	Not integrate to host-cell
		Intravenous injection	Dilution due to cell division
		Intracerebral ventricular injection	Labor intensive and special equipment in need
**RETROVIRUSES**			
Lentivirus	9 kb	Stereotaxic injection	Safety concerns: insertional mutagenesis, Impact both dividing and none dividing cell
Non-lentivirus			Not traverse the nuclear membrane
			Impact actively dividing cells
Adenovirus	Roughly 35 kb	Stereotaxic injection	Require a certain minimal amount of gene
			Acute inflammation
			Ad product is time consuming

### Non-viral mediates delivery methods

In contrast to the viral vectors, non-viral mediates delivery methods do not rely upon the evolved capabilities of viruses to insert genetic material. The non-viral vector mediates gene transfection were no longer limited by the capacity of gene material. Non-viral mediates delivery methods including electroporation, microneedles, polyethylenimine (PEI), polymersomes, polyspermine, the use of branched molecules and cationic lipid-mediates delivery (Boado, 2007; De Vry et al., 2010; Duan et al., 2012; Xiang et al., 2012; Ma et al., 2013; Chen et al., 2014, 2016a,b; Ge et al., 2014a,b; Peng et al., 2014; Che et al., 2016; Song et al., 2016).

Electroporation is a physical transfection method. It created temporary pores via electrical pulse in cell membranes through which substances like nucleic acids can pass into cells. Electroporation could almost target all the cells types *in vitro*, including mammalian cells and bacteria (Ding and Fan, 2016). Electroporation is time saving, labor saving, and cut the cost to a low level. It shows less toxicity and immunogenicity (De Vry et al., 2010; Yang B. et al., 2012; Gee et al., 2015).

Cationic lipid-mediated delivery is more rapid than viral ones. It can pack large gene material. The expression onset of RNA is much faster than that with DNA, for it needn't transit the nuclear membrane. RNA delivery is good at short-term transient gene expression, for its quick onset (Hecker, 2016). Comparing with RNA, DNA is benefit for it's longer duration and higher mean level of expression, although the onset of which is slower than RNA (Bauer et al., 2002).

The branched molecules, such as polyethylenimine (PEI), dendrigraft poly-L-lysine and chitosan are kind of physical power (Corso et al., 2005; Trapani et al., 2011; Peng et al., 2014). Which force the cell to take up the exogenous gene materials they packed. The common advantage of them is less cytotoxicity and high transfection rates, some can deliver from the ventricular which simplify the procedure. Further more, some scientists even use them to accomplish non-invasive imaging for tracking (Batrakova et al., 2007; Liu et al., 2013). Table 1b is the sum of alternative of viral vector-mediates gene transfection.

**Table 1B:** **Alternative of viral vector-mediates gene transfection**.

**Non-virus**	**Capability**	**Delivery method**	**Benefit compare to viral vector-mediates gene transfection**
Electroporation	>10 kb	–	Do not integrate/time saving/cost low
			Less toxicity/less immunity
			Higher efficiency/bigger capacity
**CATIONIC LIPID MEDIATES DELIVERY**			
DNA	Unlimited		Longer duration
		Stereotaxic injection	Higher mean level of expression per cell
RNA			Unnecessary of transit across the nuclei membrane
			Onset of expression is faster than DNA
			Preferable in some clinical applications
**POLYETHYLENIMINE**			
	Unlimited	Bone-marrow derived macrophage system	Less cytotoxicity
			Increased cellular uptake and stability
			Non-invasive imaging for tracking
		Ventricular delivery	
**DENDRIGRAFTED POLY-L-LYSINE**			
	Unlimited	Ventricular delivery	Cross the BBB by specific receptor mediated transcytosis
			Targeted nanoparticles could accumulate in brain more efficiently
**CHITOSAN WITH GRAFT WITH PEI**			
	Unlimited	Stereotaxic injection	Improved water solubility
			Low toxicity
			High transfection efficiency

## Gene therapy for Parkinson's disease

### Gene therapy for delivery of neurotrophic factors

Neurotrophic factors, such as glial cell-derived neurotrophic factor (GDNF), neurturin are secreted proteins that play regulatory roles in the development, survival and maintenance of the nervous system (Hegarty et al., 2014). While the neurodegeneration progressing, the progresses in the constructor of viral vectors for gene transfer make gene therapy a realistic form for PD treatment.

#### Gene therapy of GDNF

GDNF has attracted a lot of attention for its neurogenerative and neuroprotective effects. *In vitro*, it promoted the survival of cultured ventral midbrain dopaminergic neuron (Lin et al., 1993; Clarkson et al., 1997; Eggert et al., 1999). Then followed some studies supporting the positive effect of GDNF expression on nigrostriatal degeneration and related motor symptom in PD model animals (Eslamboli et al., 2003, 2005). Considering that the majority of the less advanced PD patients are still fully responsive to L-dopa therapy, they might not accept the irreversible measure. But very early interference of gene therapy after diagnoses may have benefits on them. Some researchers use mifepristone and AAV-5 vectors that expressing GDNF to establish an intermittent and reversible mode to control the expression of GDNF, in this system, mifepristone was used as a gene switch to induce a transient impact on expression. Animals that were injected with the constitutively expressing GDNF vectors showed a long-term and stable improvement on GDNF level, while the animals that were injected mifepristone to induce short–term expression also showed a robust but short-term improvement (Kirik et al., 2016). These result shows that the new intermittent, reversible methods also have a significant and stable impact on the gene expression while compared with the traditional irreversible way. For those less advanced PD patient, it means that they will have a more safe and useful choice to prevent neurodegeneration. Some scientists use MPTP-treated rhehus monkeys and aged monkeys with parkinsonian symptom as the model animals, both groups use AAV2-GDNF vector injected the putamen, the result turns out that AAV2-GDNF enhanced the locomotor activities and increased the dopaminergic terminals in the putamen (Eberling and Kells, 2009; Johnston et al., 2009). Although there is lot of benefits in the GDNF gene therapy, significant weight loss induced by nigral GDNF expressing is disturbing. The GDNF therapy needs more understanding and further development at every level. Table 2a is the sum of gene therapy of GDNF.

Table 2A**Gene therapy of GDNF**.

**Table:** 

**Model animal**	**Study duration**	**Target area**	**Interference of gene therapy**
6-OHDA lesioned rats	17 weeks	Striatum	Constitutive GDNF expression AAV vector
			Mifeprestone induced once
			Mifeprestone induced twice
MPTP-rhesus monkeys monkeys	12 months	Putamen	AAV2-GDNF vector
aged naïve monkeys	6 months	Putamen substrantial nigra	AAV2-GDNF vector

**Table:** 

**Interference of gene therapy**	**Restoration of the motor control**		**Impact on dopaminergic pathway**
	**Cylinder test**	**APO-induced rotation**	
Constitutive GDNF expression AAV vector	From 20 to 38%right forepaw use	Stable reduction of the Apo induced rotation by about 80%	DA level almost completely restored than the contralateral hemisphere
Mifepristone induced once	From 20 to38% right forepaw use, then declined to 25% at 15 weeks after lesion	A reduction by about 50 ~ 60% which lasts 5 weeks, and then increased at 7 weeks	Not significantly increased at the end of the experiment
Mifepristone induced twice	From 20 to 35%right forepaw use	Rotation behavior was stable reduced over the time course of study	Significant lower than those achieved by constitutive GDNF expression
AAV2-GDNF vector	Enhanced locomotor activity		Increased density of Dopaminergic terminals in the putamen
AAV2-GDNF vector	An increase of basal locomotor activity		Small increase of AADC activity

#### AVV-neurturin

The gene of neurturin is a member of the GDNF gene family. Neurturin (NTN) can rescue dopaminergic neurons damage (Biju et al., 2013). 12 PD patients receive an injection of AAV-NTN and their symptomatic syndrome showed a significant improvement, although the dopaminergic terminal showed no significant increases in PET imagination (Marks et al., 2008). However, in an effort to validate the efficacy in the former trial, researchers launched a randomized, sham surgery-controlled, double-blind clinical trials which include 58 PD patients (Marks et al., 2010). There is no significant improvement observed in the NTN-treated patients until it comes to the 18 months, while with the phase I trial only spent 12 months to meet its endpoint. The outcome of the phase II trial is modest, but significant. In the postmortem analysis of 2 deceased patients demonstrated NTN expression in putamen rather than substantial nigra, shows that there is still limitations in the retrograde transport of AVV vector. In the purpose to solve the retrograde transport problem, researchers use a more direct way, they directly target the substantial nigra and injected higher dose to putamen, and provide longer clinical follow-up. The result let them down, the clinical trial only demonstrated the tolerability of treatment with CERE-120, the drug was delivered to both parts of substantia nigra pars compacta and putamen without safety complication in a 2-year follow-up (Bartus et al., 2014), but in the subsequent phase II, it failed to show its efficacy. It didn't meet the primary endpoint they expect (off-state motor UPDRS Scores). The result of NTN-treatment was disappointing.

### Gene therapy for modulating the synthesis of neurotransmitter

#### Adeno-associated virus (AAV)-glutamate decarboxylase (GAD)

We all know that in PD, the loss of the dopaminergic neuron can result the imbalance in basal ganglia circuitry, for example, the subthalamic nucleus (STN) will receive less GABAergic input. In activation of neurons in the STN of the MPTP-treated monkey model, animals had shown ameliorated parkinsonian motor symptoms (Wichmann et al., 1994; Guridi et al., 1996). The therapeutic effect is consistent with the notion STN hyperactivity or dysfunction in PD (Bergman et al., 1994). Then some researchers want to validate the therapeutic and neuronal effect of blocking STN, they performed microinjection of local anesthetic lidocaine and muscimol, (lidocaine has the none selectively blocking axonal fibers of passage as well as neurons, while muscimol selectively inhibited the cell body of neuron.), this study shows that microinjections of pharmacological blocking agent in the STN of PD patients result in a transient anti-Parkinsonian effect (Levy et al., 2001). Then a gene therapy approach was investigated for increasing GABAergic tone in STN. Some scientists use an AAV vector to transfer the gene of GAD to STN. As the rate-limiting enzyme for the synthesis of GABA, this approach could increase the level of GABA in STN and improve the motor symptoms of PD. In the research of the subsequent open label, safety and tolerability clinical trial, they use viral vectors (AAV-GAD) injected 12 PD patients' unilateral subthalamic. The patients were divided into 3 groups; the main efficacy measure was UPDRS. Significant improvement was seen 3 months after gene therapy and persisted up to 12 months. The conclusion of the trial is that AAV-GAD gene therapy of the subthalamic nucleus is safe and well tolerated by patients with advanced PD (Kaplitt et al., 2007).

#### Aromatic amino acid decarboxylase (AADC) and tyrosine hydroxylase/aromatic amino acid decarboxylase/guanosine triphosphate cyclohydrolase (AADC-TH-GCH) gene therapy

The beneficial effect of oral administration of L-dopa will soon be complicated by motor symptoms. Olanow et al. find that continuous dopamine stimulation might counter balance these long-term effects of drug (Olanow et al., 2000, 2006a,b). Based on these theories, researchers focused on increasing dopamine level through enhancing the chemical synthesis of dopamine from levodopa. The conversion of levodopa to dopamine needs the enzyme acromatic L-amino acid decarbocylase (AADC) (Christine et al., 2009; Muramatsu et al., 2010). With the PD advancing, activities of AADC diminished, this further limiting dopamine level and resulted a larger need for the dosage of levodopa (Witt and Marks, 2011). The transduction of AADC gene to intrinsic striatal neurons could enhance the synthesis of dopamine and might improve the dopamine level in brain. The continuous existence of DA might reduce the need of levodopa in advancing PD (Allen and Feigin, 2014), and with the reduction of levodopa, the side effect like LIDs might be alleviated. A phase I clinical trial of gene transfer of AAV mediated gene delivery of AADC into putamen of 6 PD patients was launched. using multiple measures, including UPDRS, motor state diaries and PET trace for AADC, 6 months after surgery, the off-state of motor function was improved by 46% based on UPDRS scores, PET shows a 56% increase in FMT activities, both effect last 96 weeks (Muramatsu et al., 2010).

The TH/AADC/GCH is a triple gene approach. The production of dopamine needs these 3 genes to exert their function. The TH and GCH catalyze the dietary tyrosine convert to levodopa, AADC turns the levodopa to dopamine, the triple gene vectors just like a molecular machinery for manufacturing dopamine (Azzouz et al., 2002; Jarraya et al., 2009). The trisictronic lenti-vector aims to improve the dopamine level in striatal not only by restoring AADC activity, but also by further increasing endo-levodopa in both dopaminergic and non-dopaminergic neurons (Azzouz et al., 2002; Jarraya et al., 2009). This approach brings the good news that it is safe and tolerable, modest improvement in UPDRS-motors of scores was seen at 6 to 12 month (Oxford biomedica).

Although the DA-synthetic strategies have yield encouraging results, but take the small numbers of samples and the likelihood of placebo effect into consideration, further study was awaited (Christine et al., 2009; Palfi et al., 2014).

### Gene therapy for modulate proteins in PD

As we know, 6-OHDA-lesioned Parkinsonian rats had a multiple protein- and brain region-specific changes. The dysregulation of DA receptors induced by the 6-OHDA lesioning is believed to underlie PD pathology and LID. Ahmed et al had reported that the G-protein-coupled receptor kinases (GRKs) had abnormal expression level and the subcellular distribution in the basal ganglia of the 6-OHDA-lesioned parkinsonian rats, and these changes were not normalized by L-dopa treatment. Furthermore, they found that the up-regulation GRK6 (a subtype of GRKs) is similar in both 6-OHDA-lesioned PD rats and in MPTP- monkeys (Ahmed et al., 2008). It is know that G protein–coupled receptor kinases (GRKs) control desensitization of DA receptors. They set out to use a lentiviral system to increase the availability of GRK6 in the purpose to facilitation of the receptor desensitization, this measure ameliorates dyskinesia and increase duration of the antiparkinsonian action of L-dopa. The lentivirus-mediated overexpression of GRK6 is a promising method to alliviating the motor complications of PD (Ahmed et al., 2010, 2015). Recently, a lot of studies demonstrated that glycogen synthase kinase-3 activities was up-regulate while treated with high dosage of L-dopa. It was also reported that in MPTP-treated monkeys, increased activation of GSK-3β was combine with the LID (Morissette et al., 2010). It's believed that GSK-3β was involved in the development of LID. Inhibiting the expression of GSK-3β via RNA interference might also be a possible approach to reduce the motor symptoms.

### RNA interference-based therapy: a promising class of drug therapy for PD

As a very important and resent part of gene therapy, RNA interference (RNAi) is a very useful experimental tool for diagnosis and therapy in PD. RNAi could almost silence any selected gene, via which, we can conclude the genetic reason of a certain disease (Dykxhoorn and Lieberman, 2005; Waseem, 2006; Jadiya et al., 2015; Chaudhuri et al., 2016). When a long double-strand RNA (dsRNA) produced by an introduced transgene encounter a cell, an enzyme called dicer cut the long dsRNA into small pieces named siRNAs, and then siRNA induced silencing complex (RISC) picked the complementary sequence to the target gene for silencing, so that no protein is produced (Novina and Sharp, 2004; Konno et al., 2016).

### RNA interference and gene of SNCA, PINK, and Parkin

Scientists have done a lot of experiments on RNA interference of SNCA (Nagarajan et al., 2015; Takahashi et al., 2015). *In vitro*, they use polyethylene glycol-polyethyleneimine as a vector for α-synuclein siRNA delivery to PC12 cell. The polyethylene glycol-polyethyleneimine (PEG/PEI) siSNCA complex were well developed and with low cytotoxicity. It shows high transfection efficiency, suppressing the SNCA mRNA expression and preventing cell from death via apoptosis induced by 1-methyl-4-phenylpyridine (mpp+) (Liu et al., 2014). *In vivo*, down-regulations of α-synuclein shows a promising future for synucleinopathies in PD. Human SNCA gene silencing with AAV-micro30-hSNCA in rat substantia nigra could have benefits on forelimb behavior and substantia nigra dopaminergic neuron loss, but these positive effects was compromised by the inflammation which was triggered by the co-expression of either silencing vector and the reduced Tyrosin hydrocylase-immunoreactive expression (Bonin et al., 2004; Khodr et al., 2014). Table 2b is the sum of *In vivo* and *In vitro* study of *RNA* interference therapy for PD.

Table 2B*****In vivo*** and ***In vitro*** study of ***RNA*** interference therapy for PD**.

**Table:** 

**Complexes**	**Model animal**	**Level of SCNA In brain**	**Restoration of motor**
**I:** ***In vivo***			
shSCNA_T1-2	PD model flies	Decreased	Motor dysfunction increased
			Depending upon the reduction of SNCA
AAV-mir30-hSCNA		Decreased	Protect against the forelimb deficit

**Table:** 

**Complexes**	**Model cell**	**Level of SNCA**
**II:** ***In vitro***		
siSNCA_T1-2	PD patients' fibroblast	Decreased endogenous SCNA to an half level
		The half level of SCNA is similar to that in normal
siSCNA4	SH-SY5Y cell	7.69-fold reduction of SCNA mRNA
		2.43-fold reduction of SCNA protein
siSCNA1		1.59-fold reduction of SCNA mRNA
		1.51-fold reduction of SCNA protein
PEG-PEI/siSCNA	PC12	Suppress SCNA mRNA expression

Some other researchers use the RNAi technique help to form a specific biomarker to detect sporadic PD. Some researchers even use skin fibroblast from PD patients and the control lines with knock-down of PINK1, to investigate the expression changes of SCNA, they reveal that the expression changes detected from the two lines of cell may have the potential to see as a biomarker that allow physician to diagnosis objective PD in an non-invasive way (Hoepken et al., 2008). Mutations in PINK1 associated with early onset autosomal recessive PD, loss of it also caused CI deficiency (Zhang et al., 2013; Ng et al., 2014; Min et al., 2015). Furthermore, PINK1 together with another PD gene, Parkin, co-regulates the mitochondrial morphology and mitophagy (Ivatt et al., 2014).

## Conclusion

In recent years, PD and levodopa-induced dyskinesia remains to be a hard nut to crack. While L-dopa replacement therapy trapped in a dilemma, gene therapy showed its vigorous vitality in biology research. Gene therapy has made several heart-stirring progresses: Maddalena et al. (2013) and Tereshchenko et al. (2014) has develop the irreversible gene therapy into a controllable, and reversible manner via the mifepristone as a gene inducer, it make the gene therapy became useful even in the less advanced PD patient, this method can also promoted to other neurodegenerative disease while treated with gene therapy. Ahemd et al. had use the lentivirus–mediated overexpression of GRK6 to desensization the DA receptors, this approach had get lot of benefits that is unattainable while directly targeting the signaling pathways. But a lot of problems still remain: the gene therapy we discussed above is directly sent the complexes to the target area, how can we avoid the inflammation. In the gene therapy of GDNF, weigh loss is a disturbing thing remained unsolved, and the development of some antibody against the GDNF was observed. With the advancing of the biology, we hope to see more help from RNAi that rescue gene deficit than is observed in diagnosis. However, applications of the RNAi technique in clinical practice still have a long way to go.

## Author contributions

JL, CX, SZ, and ZL participated in its design, searched databases, extracted and assessed studies and helped to draft the manuscript. WY conceived the initial idea and the conceptualization; WY and ZL revised the manuscript. All authors read and approved the final manuscript.

### Conflict of interest statement

The authors declare that the research was conducted in the absence of any commercial or financial relationships that could be construed as a potential conflict of interest.
